# Correlation between semiquantitative sonoelastography and immunohistochemistry in the evaluation of testicular focal lesions

**DOI:** 10.1186/s40644-014-0029-6

**Published:** 2014-10-08

**Authors:** Antonio Luigi Pastore, Giovanni Palleschi, Piero Maceroni, Giorgia Manfredonia, Domenico Autieri, Jessica Cacciotti, Barbara Sardella, Natale Porta, Vincenzo Petrozza, Antonio Carbone

**Affiliations:** 1 Department of Medico-Surgical Sciences and Biotechnologies, Urology Unit, ICOT, Sapienza University of Rome, Faculty of Pharmacy and Medicine, Latina, Italy; 2 Uroresearch Association, Latina, Italy; 3 Diagnostic Imaging Department (CADI), ICOT, Latina, Italy; 4 Department of Medico-Surgical Sciences and Biotechnologies, Histopathology Unit, ICOT, Sapienza University of Rome, Faculty of Pharmacy and Medicine, Latina, Italy; 5 Department of Pathology, S. Maria Goretti Hospital, Latina, Italy; 6 Sapienza University of Rome Faculty of Pharmacy and Medicine Urology Unit, Corso della Repubblica 79, Latina (Lt), 04100, Italy

**Keywords:** Sonoelastography, Testis, Semiquantitative elastography, Strain ratio, Neoangiogenesis, CD31

## Abstract

**Background:**

Sonoelastography is a novel and promising imaging tool, which has been applied to breast, thyroid, and prostate tissues. The aim of this study was to evaluate focal lesions of the testes with diameters of <10 mm using sonoelastography, B-mode sonography (US), and colour Doppler ultrasonography (CDU).

**Methods:**

Thirty patients who were referred to our outpatient clinics for varicocoeles, scrotal pain, scrotal enlargements, epididymitis, palpable testicular nodules, or infertility, were prospectively enrolled into this study. Ultrasound evaluations had revealed that 27 subjects had focal testicular lesions with diameters of <10 mm and 3 subjects had 10-mm spherical non-homogeneous testicular nodules. All lesions were evaluated using semiquantitative sonoelastography, and the patients underwent orchifunicolectomies. The testicular lesions were examined histopathologically. The vascularization of the lesions and the surrounding testicular parenchyma was evaluated by analysing the immunohistochemical distribution of the cluster of differentiation 31 and by calculating the vascular indices (VI). Potential associations between the strain ratios (stiffness of the lesions) and the VI were tested.

**Results:**

Analyses of the strain fields obtained using semiquantitative sonoelastography yielded different values for the masses and the surrounding tissues, which led to significant increases in the strain ratios. Sonoelastography upheld all of the diagnoses that were suspected when the patients were physically examined, when the serum markers were analysed, and after the patients had undergone US and CDU. Histopathological examinations confirmed the neoplastic characteristics of these masses. A significant inverse correlation was determined between the sonoelastographic strain ratio and the VI (Pearson correlation coefficient, r, = − 0.93; *P* < 0.001).

**Conclusion:**

Our investigation shows that semiquantitative sonoelastography may provide additional objective information to support the algorithm used to diagnose testicular lesions. This might be of crucial diagnostic importance for lesions with diameters of <10 mm, particularly if they are not palpable, are negative for serum tumour markers, and if the findings from ultrasonography and CDU are equivocal. The findings from semiquantitative sonoelastography might indicate the need for surgical exploration. Further investigations with larger numbers of patients are required to corroborate these data and to support the use of semiquantitative sonoelastography in the evaluation of testicular lesions.

## 1 Background

Ultrasonographic elastography, or sonoelastography, is a novel diagnostic modality that is under investigation for its clinical application to cancer diagnostics [1]-[4], and primarily for lesional imaging of the breast [3],[5]-[9], thyroid [10],[11], and prostate [12]-[14]. However, sonoelastography is not routinely used in the clinical environment.

An important characteristic of a tissue is its inherent elasticity, which may be altered by the pathophysiological processes associated with ageing, inflammation, and the presence of tumours. In this context, elasticity is defined as the tension (i.e. stress) needed to produce a relative change in length (i.e. strain), and it describes the amount of pressure that must be exerted on a tissue to achieve elastic deformation. In principle, elasticity can be derived from the stress and strain parameters measured in tissue structures. While strain can be computed from high-frequency echo signals, stress cannot be directly determined from tissue measurements. During ultrasonographic examinations of tissues, compressibility is a standard parameter that is considered in the differential diagnoses of lesions. A non-compressible mass detected during B-mode sonography (US) is associated with an increased likelihood of malignancy. Sonoelastography is based on strain imaging, and preliminary data on a possible role for sonoelastography in the evaluation of testicular lesions have been reported [15]-[17]. This study compared US, which is a widely used diagnostic sonographic method, with semiquantitative sonoelastography, which is a new method for visualizing the elastic characteristics of tissues, in the evaluation of testicular nodules with diameters of <10 mm. To explore the outcomes generated by this tool in greater depth, we tested the relationship between the strain ratio data obtained using sonoelastography, and the vascular indices (VI) that were determined from the immunohistochemical analysis of the testicular lesions.

## 2 Methods

### 2.1 Patients

Between March 2010 and January 2013, 30 male patients with an average age of 37 years (range 22–50 years), presented with the following findings: 9 patients had scrotal enlargements, 6 of whom had unilateral epididymitis and 3 of whom had idiopathic scrotal pain; 7 patients had palpable hard testicular nodules, 4 of whom were referred to our clinic because of varicocoeles; and 14 patients visited our outpatient clinic because of infertility issues and they did not present with any palpable lesions.

All patients were investigated using US and colour Doppler ultrasonography (CDU), which enabled the presence of testicular lesions to be confirmed, their largest diameters to be calculated, and their vascular configurations to be explored.

These lesions were then evaluated using semiquantitative sonoelastography (ElastoQ). The patients underwent orchifunicolectomies within 1 week of their diagnostic algorithms being fulfilled, which included the evaluation of tumour markers in the serum. Histopathology was performed on all of the lesions.

The study was performed according to the principles of the Declaration of Helsinki 2000. Local ethics committee approval was obtained (ASL Lt/no.12985678), and written informed consent was acquired from all patients.

### 2.2 Semiquantitative sonoelastography

Semiquantitative sonoelastography was performed while the patient rested in a supine position on a bed, using the Aplio XG Colour Doppler System (Toshiba Medical Systems Corporation, Tokyo, Japan) equipped with a 5–14-MHz linear transducer. The probe was placed on the testis while applying light pressure. At this time, the operator can see a curve representing the entity and the mode of compression in the lower portions of the display. This allows the operator to analyse the shape of the compression/decompression curve and verify that the amount of compression is optimal. Semiquantitative sonoelastography software calculates the strain ratio, with the terminal phase of decompression being selected for the calculation, because it is less dependent on the force exerted and is therefore more consistent.

During sonoelastography, the operator highlighted the nodule being evaluated, and this was referred to as the region of interest (ROI). The ROI was manually drawn to correspond with the nodule and it was at an elevation plane that corresponded with the extralesional parenchyma. The size of the ROI was defined by the operator as the area of abnormal tissue selected. Another area of the same size as the ROI was then selected within the normal tissue to enable the areas to be compared and to determine the strain ratio. Two ultrasonic images were acquired during sonoelastography: one was acquired before tissue compression by the probe and the other was obtained after tissue compression. Tissue displacement was tracked by assessing the propagation of the imaging beam, and tissue distortion was accurately measured using the dedicated software. Once the raw data had been acquired, a chromatic map was superimposed onto the US image using a colour scale that ranges from red, indicating components with the greatest elastic strain (i.e. the softest components), to blue that indicates components with no elastic strain (i.e. the hardest components).

In this study, the sonoelastographic images were matched with an elasticity colour scale and classified using the elasticity score developed by Ueno and Itoh [1]. The strain values for the oldest periods of compression or decompression were calculated automatically from the velocity profiles of all of the frames. The image of the frame in which the absolute value of the strain was at its maximum was displayed. The same operator performed all of the examinations.

To calculate the strain, we chose the optimal decompression value for each lesion. This enabled the strain ratio of the ROI to be obtained by comparing the elastic characteristics of the lesion with the elastic characteristics of the normal tissue. The echogenicity ratio was determined by comparing the echogenicity of the lesion with that of the normal tissue using the greyscale function. It is possible to define ElastoQ as a modification of the real-time method, because it allows off-line analysis of the deformation values (i.e. strain) of a mass while utilizing the raw data generated by changes in radiofrequencies before and after tissue compression. The calculation is performed by selecting an ROI within the lesion under investigation and a reference region in an area of normal parenchymal elasticity. The result is an index of deformation (i.e. strain index) that is relative to the reference value of the tissue. The strain index increases as the rigidity of the structure being examined increases. This method can also be used to determine a lesion’s degree of echogenicity by comparing it with nearby normal structures, while considering the absolute values of echogenicity expressed in the raw data.

### 2.3 Histopathological examination of the lesions

**Figure 1:**
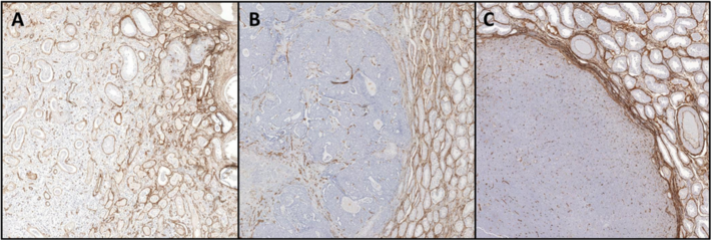
**Immunohistochemical analysis of vascularization using the CD31 antibody. (A)** Seminoma, **(B)** embryonal carcinoma, and **(C)** Leydig cell tumour (10× magnification).

All of the patients underwent orchifunicolectomy. The surgical specimens from the primary tumours were submitted for histological examination in 10% neutral-buffered formalin, and were then processed and embedded in paraffin wax. Sections were cut at 5 μm and stained with haematoxylin and eosin. Two pathologists reviewed the sections and categorized the tumours using the World Health Organization’s histological classification of testicular cancer. Immunohistochemistry was performed using the Bond Polymer Refine Detection system (Leica Biosystems, Newcastle, UK) and the cluster of differentiation (CD) 31 antibody (PECAM-1; Clone 1A10, Leica Biosystems, Newcastle, UK), at a dilution of 1:100, to determine the distribution of CD31, and hence the vascularization of the primary tumours and the surrounding testicular tissues. Two pathologists evaluated these sections at a 10× magnification (Figure 1). The results were expressed as percentages and the VI was calculated, which was derived from the difference between the vascularization of the lesion and the vascularization of the surrounding testicular parenchyma.

### 2.4 Statistical analysis

The strain ratios and VI results were statistically analysed by calculating the linear regression between the two variables. The Pearson correlation coefficient, r, was used to test for a linear association between the strain ratio and the VI. An α error below 5% was considered statistically significant. The statistical analyses were performed using IBM® SPSS® version 18.0 (IBM, Armonk, New York, USA).

## 3 Results

When examined by US, 27/30 patients had subcentimetre hypoechoic lesions and 3 patients had 1-cm non-homogeneous spherical lesions. Only 7/30 lesions showed clear evidence of increased vascular flows when examined by CDU. The findings in these 7 patients combined with the positive lesional palpations detected during the physical examinations, which raised suspicions about the presence of neoplastic disease, prompted us to assess all of the patients for the presence of the following tumour markers: alpha-fetoprotein, carcinoembryonic antigen, human chorionic gonadotropin, and lactate dehydrogenase. Only 4/30 patients were positive for these tumour markers.

**Figure 2:**
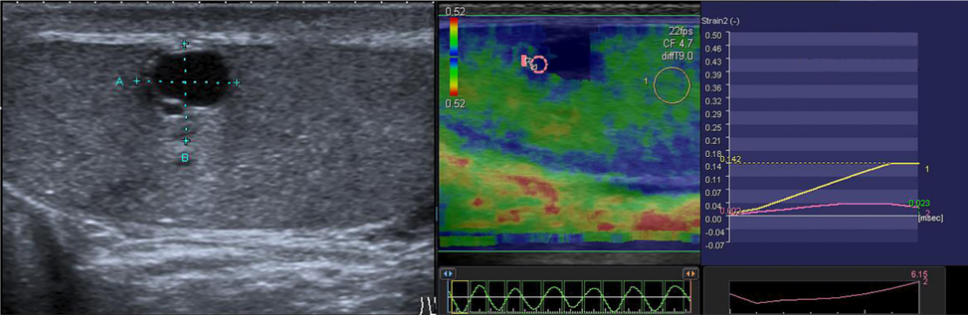
Cystic nodule with high SR (6.15) in peripheral solid portions (6 mm) in a 22 years old patient.

**Figure 3:**
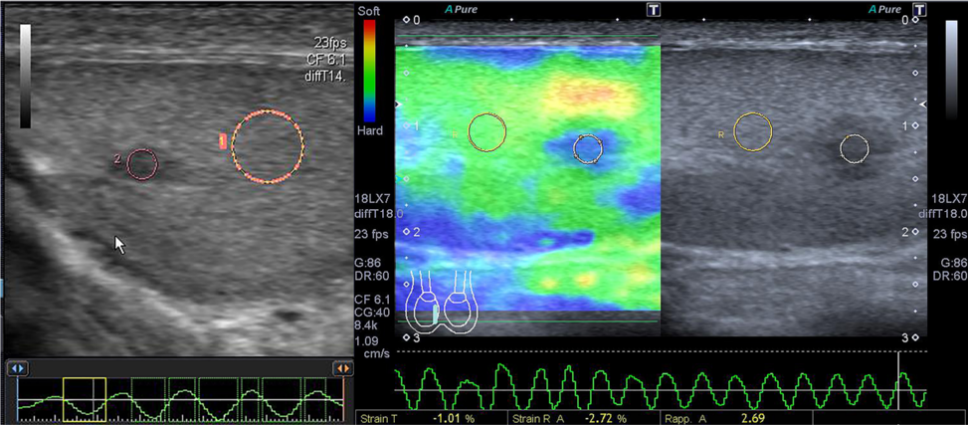
Solid nodule (7 mm) with high SR (2.69) discovered during ultrasound scan in a 36 years old patient with primary infertility.

**Figure 4:**
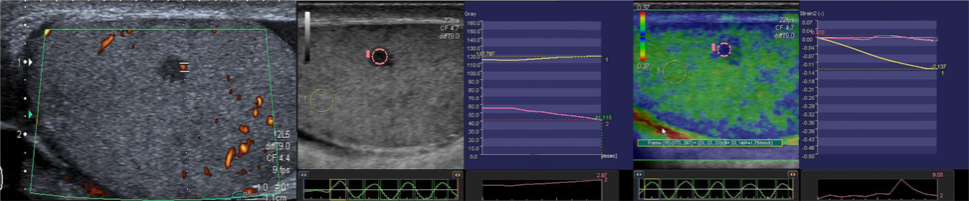
Solid nodule (5 × 4 mm) with modest intralesional vascularity and extremely high SR (23.2) in 42 years old patient.

**Figure 5:**
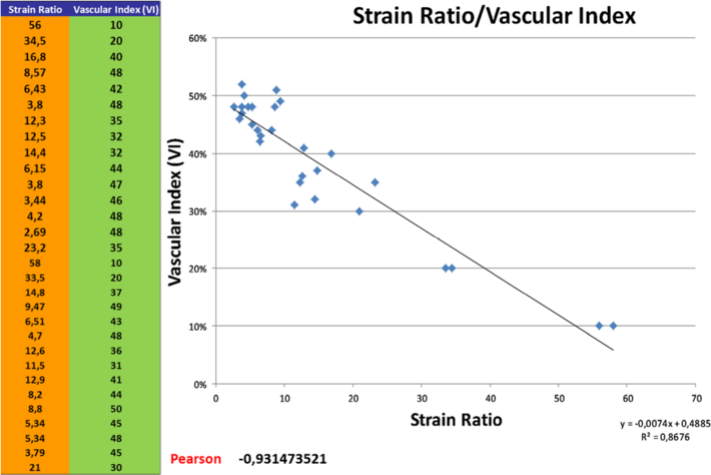
**The significant inverse correlation between the strain ratio (stiffness of the lesion) and the vascular index (VI) relating to neoangiogenesis within the tumour.** This could be interpreted as the ability to quantify angiogenesis using sonoelastography.

Semiquantitative sonoelastography provided data that supplemented those generated by US and CDU. Specifically, this tool demonstrated that the normal testicular tissue and nodules had different elastographic patterns, with the latter showing stiffer sonoelastographic signals, particularly at the perimeters of the masses (Figures 2, 3, 4), which was suggestive of pathological tissue, and has been described in previous reports [5]. These findings corroborated our suspicions about the neoplastic origins of these lesions; consequently, we performed orchifunicolectomies. The histopathological examinations determined the presence of 18 seminomas, of which 15 were classic, 2 were anaplastic, and 1 was spermatocytic, 3 mixed-germ cell tumours, containing embryonal carcinomas, yolk sac tumours, and seminomas, 2 embryonal carcinomas, and 7 Leydig cell tumours. The 2 patients with embryonal carcinomas and 2/3 individuals with mixed tumours were positive for the serum tumour markers. The vascularization of the primary tumours and the surrounding testicular parenchyma was evaluated immunohistochemically using the CD31 antibody, and this was used to calculate the VI. The Pearson correlation coefficient showed a significant inverse correlation between the sonoelastographic strain ratio (stiffness of the lesion) and the VI (r = − 0.93; *P* < 0.001) (Figure 5). The clinical features of the patients, together with their histopathological results, strain ratios, and VI are summarized in Table 1.

**Table 1:** Clinical features of the patients, their histopathological results, strain ratios, and vascular indices

**Pt**	**Age**	**Clinical features**	**Serum tumor markers (AFP, LDH, βhCG) at diagnosis**	**Lesion size (mm)**	**Histotype**	**Adjuvant therapy**	**Strain ratio**	**Vascular index**
1	41	Scrotal pain	Positive (βhCG)	5.7	Embryonal carcinoma	Chemotherapy	56	10%
2	50	Epididymitis, cryptorchidism	Negative	8.6	Classic seminoma	NO	34,5	20%
3	32	Infertility	Positive (βhCG, AFP)	7.9	Embryonal carcinoma	Chemotherapy	16,8	40%
4	35	Infertility, palpable testicular node	Negative	10.4	Classic seminoma	NO	8,57	48%
5	34	Epididymitis	Negative	5.6	Classic seminoma	NO	6,43	42%
6	35	Infertility, palpable testicular node	Negative	10.3	Classic seminoma	NO	3,8	48%
7	35	Infertility	Negative	5.9	Classic seminoma	NO	12,3	35%
8	37	Infertility	Negative	5.8	Leydig cell tumor	NO	12,5	32%
9	38	Infertility	Positive (AFP)	6.5	Mixed germ cell tumor	Chemotherapy	12,5	41%
10	22	Varicocele, palpable testicular node	Negative	6.1	Classic seminoma	NO	6,15	44%
11	28	Varicocele	Negative	7.6	Mixed germ cell tumor	Chemotherapy	3,8	47%
12	41	Infertility	Negative	7.8	Leydig cell tumor	NO	3,44	46%
13	49	Palpable testicular nodule	Negative	9.6	Leydig cell tumor	NO	4,2	48%
14	36	Infertility	Negative	6.9	Anaplastic seminoma	Radiotherapy	2,69	48%
15	42	Infertility	Negative	5.1	Leydig cell tumor	NO	23,2	35%
16	38	Infertility, cryptorchidism	Negative	7.9	Spermatocytic seminoma	NO	58	10%
17	33	Epididymitis	Negative	8.6	Classic seminoma	NO	33.5	20%
18	24	Varicocele, palpable testicular node	Negative	10.2	Classic seminoma	NO	14.8	37%
19	29	Varicocele	Negative	7.4	Classic seminoma	NO	9.47	49%
20	37	Infertility, cryptorchidism	Positive (AFP)	6.5	Mixed germ cell tumor	Chemotherapy and RLND	6.51	43%
21	43	Epididymitis	Negative	4.3	Leydig cell tumor	NO	4.7	48%
22	42	Infertility	Negative	4.6	Classic seminoma	NO	12.6	36%
23	46	Scrotal Pain	Negative	5.4	Leydig cell tumor	NO	11.5	31%
24	22	Varicocele, palpable testicular node	Negative	8.8	Classic seminoma	NO	12.9	41%
25	36	Infertility	Negative	6.3	Classic seminoma	NO	8.2	44%
26	36	Epididymitis, cryptorchidism	Negative	5.6	Anaplastic seminoma	NO	8.8	50%
27	33	Infertility	Negative	6.7	Classic seminoma	NO	5.34	45%
28	39	Scrotal pain	Negative	4.3	Classic seminoma	NO	5.34	48%
29	22	Varicocele, palpable testicular node	Negative	8.7	Classic seminoma	NO	3.79	45%
30	36	Epididymitis	Negative	5.6	Leydig cell tumor	NO	21	30%

*Abbreviations: AFP* alpha fetoprotein, *LDH* lactate dehydrogenase, *βhCG* beta human chorionic gonadotropin, *RLND* retroperitoneal lymph node dissection.

## 4 Discussion

Testicular masses can be difficult to diagnose, particularly when they have a diameter of <1 cm and/or are not palpable. Palpation provides subjective data, but physicians need instrumental support to make informed decisions about therapy when the lesion is small. Evaluations using US combined with CDU are commonly used to characterize testicular lesions, but these evaluations do not provide any information about the internal consistency of the masses. This information might help to corroborate diagnostic assessments, especially when masses with small diameters are found incidentally. Sonoelastography can be used to evaluate the mechanical elastic properties of soft tissues, which are determined by their macromolecules and structural organization. This is the first report describing the use of semiquantitative sonoelastography to investigate subcentimetre testicular masses, which could be promising potential targets for this new imaging technology. Indeed, the information obtained through sonoelastography in this study has contributed to the diagnostic evaluation of testicular lesions, because it has given an insight into their atypical compositions and it has emphasized the need for a high level of clinical suspicion about malignancy in the context of subcentimetre testicular tumours. Our data concur with those from previously reported studies [15]-[17] and they ratify the potential role of sonoelastography in the evaluation of testicular nodules.

Sonoelastography evaluates the radiofrequencies that arise from structures before and after they are subjected to manual or mechanical compression. Manual compression and mechanical compression are the different sonoelastographic techniques used to cause deformation of the structures under investigation. The intensity of the change in the radiofrequency of the ultrasound pulse rises as the severity of the deformation and the elasticity of the tissue increase. The evaluation of the strain or deformation induced by manual compression is a clinically proven method for the evaluation of tumours [1]-[4],[11]-[13].

This technique does not require any additional hardware, and it only requires the software associated with the sonoelastographic system [11]. The data obtained using this system are shown on the monitor in real time with a colour map superimposed onto the ultrasonographic image that arbitrarily encodes the structure’s elasticity characteristics. The data are expressed in terms of the tissue deformation capacity of the structure in question.

Sonoelastographic results can be affected by several factors including coarse calcifications, cysts, and out-of-plane displacements, which can compress the lesion under examination. However, the testis is mobile and it is not adjacent to any bony structures. Since real-time sonoelastography is based on the superimposition of polychromatic maps of elasticity onto 2-dimensional images, elasticity is determined subjectively and nodular lesions are scored from E1 to E5 in accordance with the mapping scheme defined by Ueno and Itoh [1]. Previous studies of real-time sonoelastography have demonstrated that scores of E4 and E5 have high predictive values for malignancy, the ability of the score to predict malignancy is independent of nodule size, and that high specificities and sensitivities are maintained for lesions of <1 cm diameter [18].

In our study, we sought to substantially reduce the subjectivity associated with evaluating the colour maps in real time by replacing the classification system with a numerical parameter, that is, the strain ratio, which is not operator dependent, is reproducible, and enables the elasticity of lesions to be given unique classifications. To ensure the objectivity of the assessment, we also attempted to define a cut-off value that would differentiate between likely benign nodular lesions and likely malignant lesions, and to add a numerical parameter that would help guide the next clinical step, for example, clinical monitoring with or without treatment, or surgical excision. This was possible with the semiquantitative sonoelastography system, because it expresses the deformation capacity of the structure under investigation relative to the normal (reference) tissue as an absolute value or as a strain ratio. Therefore, semiquantitative sonoelastography represents a modification of the real-time method and enables the off-line analysis of a nodule’s strain values by analysing variations in radiofrequencies using the raw data. The calculation is performed by selecting an ROI within the nodular formation and a reference region in an area of normal parenchymal elasticity. The result is an index of deformation (i.e. a strain index or a coefficient of elasticity) that can be compared with the organ’s reference values, and that rises as the rigidity of the structure being examined increases. Using this method, the coefficient of echogenicity can also be determined by comparing the echogenicity of the lesion with that of nearby normal structures, while taking into account the absolute values of echogenicity expressed in the raw data.

In a preliminary study, Grasso et al. [19] used sonoelastography to evaluate various pathological conditions of the scrotum and testes. They evaluated 41 men with scrotal pain, painless enlargements of the scrotum, testicular nodules, and infertility. The authors concluded that sonoelastography could provide additional valuable information, because compared with other sonographic methods, it provided better clarity in patients who had solid testicular lesions with diameters of <10 mm. Grasso et al. [19] highlighted the advantages of the technique when using it to differentiate between neoplastic masses that exhibit homogeneous hardening and haematomas that are characterized by non-homogeneous sonoelastographic patterns. Other authors support the use of sonoelastography for the evaluation of testicular masses. For example, Magarelli et al. [15] reported that sonoelastography helps to elucidate those ambiguous soft-tissue lesions that are identified using conventional ultrasonographic techniques. Furthermore, Aigner et al. [16] evaluated 50 individuals affected by testicular masses using sonoelastography. They found that 34/50 of the lesions were tumours, and they reported that sonoelastography can provide additional information about patients who have indeterminate ultrasonographic findings, because of its higher specificity compared with other sonographic methods. A recent publication by Goddi et al. [17] reported the results of 88 consecutive testicular evaluations using sonoelastography. In their experience, real-time tissue elastography gave 87.5% sensitivity, 98.2% specificity, a 93.3% positive predictive value, a 96.4% negative predictive value, and 95.8% accuracy in differentiating between malignant and benign lesions. Most of the malignant lesions within this case series (87.5%) showed a stiff elastographic pattern, leading the authors to conclude that real-time tissue elastography differentiates between benign and malignant testicular lesions more effectively [17].

Our experience demonstrates that semiquantitative sonoelastography is a viable option for the evaluation of testicular nodules, and that it might offer an instrumental option that could support conventional ultrasonographic approaches in the diagnostic algorithm for testicular nodules, especially in the diagnosis of ambiguous and non-palpable lesions. The findings from this study suggest that tumours emit signals indicative of stiffer tissues, especially in the region around a tumour’s perimeter. Given that this was found in all of the tumours examined, signals indicative of stiffer tissues within suspicious echographic areas of the testis might be considered reliable indicators of pathological tissues. In the current study population, only 4 patients were positive for tumour markers in their sera, 27 individuals had subcentimetre lesions, and only 7 individuals had palpable masses. Hence, not all of the subjects had definitive diagnoses of neoplastic disease.

The data generated by semiquantitative sonoelastography provide further evidence that the lesions discovered during the physical examinations, US, and CDU were pathological, which prompted surgical treatment. Furthermore, the immunohistochemical analysis undertaken in our study demonstrated a linear inverse correlation between the strain ratio values and the VI. It could be postulated that this inverse correlation is an expression of the lesions’ diminished consistencies and greater intralesional vascularity. This finding could be further interpreted as hypervascular tumours having lower levels of consistency than hypovascular tumours. Hence, sonoelastography could quantify the magnitude of intralesional microcirculation or neoangiogenesis, and substantiate the consistency of testicular masses. Together, these add important elements to diagnostic and prognostic assessments. Given that the main limitation of the present study was the small number of patients, further investigations with larger numbers of patients are required to corroborate these data and to support the use of semiquantitative sonoelastography in the evaluation of testicular lesions.

## 5 Conclusion

Among the patients studied, we found that semiquantitative sonoelastography provided valuable additional objective information about solid testicular lesions, which was of crucial diagnostic importance, and that it confirmed the need for surgical exploration, particularly when the tumours were <10 mm in diameter. The results from this patient series indicate that semiquantitative sonoelastography is a promising adjunctive modality for evaluating the consistency and microvascularity of testicular lesions. However, future studies using larger numbers of patients are necessary to validate these data.

## Abbreviations

CD: Cluster of differentiation

CDU: Colour doppler ultrasonography

ROI: Region of interest

US: B-mode sonography

VI: Vascular indices

## Competing interests

The authors declare that they have no competing interest.

## Authors’ contributions

All authors contributed equally to drafting the manuscript. All authors read and approved the final version of the manuscript.
